# Exploring the Significance, Extraction, and Characterization of Plant-Derived Secondary Metabolites in Complex Mixtures

**DOI:** 10.3390/metabo14020119

**Published:** 2024-02-11

**Authors:** Ruchi Barthwal, Rohit Mahar

**Affiliations:** Department of Chemistry, Hemvati Nandan Bahuguna Garhwal University (A Central University), Srinagar Garhwal 246174, Uttarakhand, India; ruchi0426barthwal@gmail.com

**Keywords:** liquid chromatography, mass spectrometry, natural products, nuclear magnetic resonance, plants, secondary metabolites

## Abstract

Secondary metabolites are essential components for the survival of plants. Secondary metabolites in complex mixtures from plants have been adopted and documented by different traditional medicinal systems worldwide for the treatment of various human diseases. The extraction strategies are the key components for therapeutic development from natural sources. Polarity-dependent solvent-selective extraction, acidic and basic solution-based extraction, and microwave- and ultrasound-assisted extraction are some of the most important strategies for the extraction of natural products from plants. The method needs to be optimized to isolate a specific class of compounds. Therefore, to establish the mechanism of action, the characterization of the secondary metabolites, in a mixture or in their pure forms, is equally important. LC-MS, GC-MS, and extensive NMR spectroscopic strategies are established techniques for the profiling of metabolites in crude extracts. Various protocols for the extraction and characterization of a wide range of classes of compounds have been developed by various research groups and are described in this review. Additionally, the possible means of characterizing the compounds in the mixture and their uniqueness are also discussed. Hyphenated techniques are crucial for profiling because of their ability to analyze a vast range of compounds. In contrast, inherent chemical shifts make NMR an indispensable tool for structure elucidation in complex mixtures.

## 1. Introduction

Phytochemicals are chemical constituents produced naturally by plants via biosynthetic processes that are present in nature [1]. These phytochemicals are often called natural products, secondary metabolites, or phytoconstituents [2]. These chemicals play an important role in the biological activity of plants, such as survival in adverse conditions and defense mechanisms against pathogens or predators [3,4]. Due to the diverse chemical properties of natural products, these are being used as therapeutic regimens against various human diseases [2,5]. Many phytochemicals have demonstrated biological properties such as anti-inflammatory and antioxidant activity and minimizing the risk of cardiovascular disease, cancer, type 2 diabetes, liver diseases, cataracts, and progression in Alzheimer’s disease [1,6,7,8].

The medicinal value of natural products is immense, but the extraction of these from plants is a herculean task [9,10]. Some of the most important natural products obtained from plants are terpenoids, steroidal glycosides, alkaloids, and phenolics such as tannins and flavonoids [11,12]. Plant-derived natural products have been the most successful candidates for potential drug lead molecules. However, their implementation in drug discovery and development has seen a decline in interest due to difficulties in isolation and characterization and in finding new chemical entities. Nevertheless, natural products could provide unique structural diversity, offering immense opportunities for the discovery of drug lead molecules. Various methods have been developed to extract natural products from diverse matrices. The methods include maceration, percolation, Soxhlet extraction, solid-phase extraction (SPE) [13,14], supercritical fluid extraction (SFE) [15], ultrasonication-assisted extraction (UAE), and microwave-assisted extraction (MAE) [16]. Maceration is a very simple method but has a long extraction time with low extraction efficiency. In contrast, percolation is more efficient due to the continuous removal of the saturated solvent by a fresh solvent. These could be useful for the extraction of thermolabile compounds [17].

Ultrasound extraction produces high yields at a low operating temperature, enabling the extraction of thermolabile compounds. It is also efficient in the recovery and purification of active ingredients compared to microwave-assisted extraction [18]. However, it could have deleterious effects due to its use of ultrasound energy of >20 KHz on the active constituents, such as free radical formation and undesirable changes [19]. Technological advancements in chromatographic techniques have been designed to isolate and purify natural products. However, isolating every single compound from a plant is not always possible due to the requirement of highly advanced preparative chromatography and the complexity of the compounds [20,21].

The characterization of metabolites is essential to understand the chemical properties of the components present in the extracts. Liquid chromatography/gas chromatography—mass spectrometry (LC/GC-MS), nuclear magnetic resonance (NMR) spectroscopy, and X-ray crystallography are the techniques most widely used to determine the chemical structures of molecules [7,22,23]. NMR spectroscopy is a widely utilized and well-explored technique for the elucidation of the structures of secondary metabolites [24], and NMR results are unbiased as they do not depend on ionization, as is the case in mass spectrometry [25]. Additionally, NMR can be used to characterize unprecedented compounds [26] and complex secondary metabolites in complex plant extracts [7,27], but the low resolution in proton NMR spectroscopy demands high magnetic field instrumentation and 2D NMR experiments. X-ray crystallography is one of the best techniques for absolute structure elucidation but the crystallization of several classes of compounds is tedious work [28], whereas NMR requires samples in a solution state, which is an advantage over X-ray crystallography for the analysis of organic samples. Due to the high-resolution capacity and high ionizing power of the electron impact in GC-MS, the resulting fragmentation pattern could be helpful in characterizing the compounds present in the mixture [29,30], but it is not possible to derivatize all classes of volatile compounds using GC-MS [31]. There could be several, and many techniques may be utilized to characterize the compounds in a complex mixture, depending upon the source and nature of the matrices. Thus, the analysis of the secondary metabolites in a crude extract and its fractions is extremely necessary, as the biological activity of a specific class of compounds may be retained within its extracts/fractions [32].

In this paper, we demonstrate the various extraction methods for a diverse range of classes of compounds and modern analytical means of characterizing the compounds in a mixture. Additionally, the methods of extraction and characterization of the compounds are compared and critically discussed.

## 2. Experimental Design

### 2.1. Plant Material

Various materials can be used to extract natural products from plants. For example, Trapti et al. utilized the dried leaf powder of *M. koenigii* for the extraction of pyranocarbazole alkaloids [6], and Shiv Nandan et al. utilized the dried leaves of *M. koenigii* for the extraction of carbazole alkaloids [33]. Renu et al. used the dried powder of the ripe fruits of *Myristica* plants for the extraction of various types of compounds [7]. Rohit et al. utilized the roots of *Calotropis gigantea* for the extraction of oxypregnane oligoglycosides. The leaves, stems, barks, flowers, and fruits of *Alstonia scholaris* have been used for the extraction of terpenoids and alkaloids [8,22]. Singh et al. used the flowers, leaves, stems, roots, fruits, and seeds of the *Nerium oleander* to extract cardiac glycosides [34]. The stems of *Catharanthus roseus* were used to extract its phenolic content [35]. The plant materials can be ground using liquid nitrogen in a glass vial or a mortar and pestle [36]. Similarly, different plants have been used in numerous ways, and the various plant parts can be dried and ground for further extraction processes regarding the desired class of compounds or secondary metabolites.

### 2.2. Extraction Protocols of Diverse Class of Compounds

#### 2.2.1. Extraction of Alkaloids

**Figure 1:**
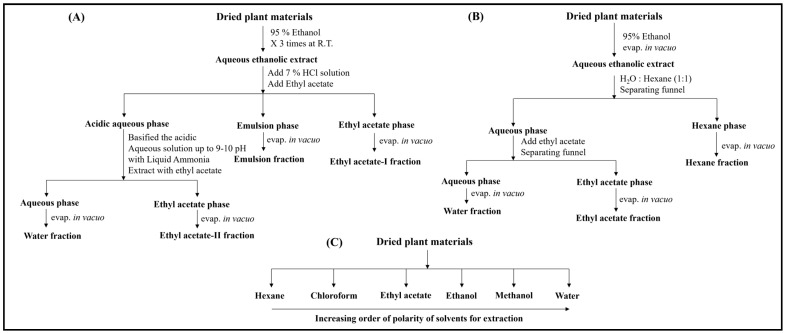
Extraction methods for phytoconstituents: (**A**) selective extraction methods for isolation of alkaloids and terpenoids using acidic and basic medium through partitioning, (**B**) extraction method for mid-polar compounds or compounds with non-polar (aglycone) and polar (glycone) moieties, and (**C**) sequential method through solvent-polarity-dependent extraction of phytoconstituents from plants.

The dried powder of the leaves of *Murraya koenigii* has been extracted with n-hexane, dichloromethane, chloroform, ethyl acetate, acetone, methanol, and ethanol, separately. This method can be specifically used to determine the extraction efficiency of different solvents for alkaloidal extraction (Figure 1C) [6]. The dried ground parts of *Alstonia scholaris* were used to extract alkaloids. Briefly, the samples were extracted with 95% ethanol (three times), filtered, and concentrated. The ethanolic extract was dissolved in 7% HCl and partitioned with ethyl acetate, resulting in ethyl acetate fraction I, which was greenish in color and might have contained chlorophyll and other non-polar compounds. The acidic solution was basified with liquid ammonia up to pH 9–10 and again partitioned with ethyl acetate, resulting in ethyl acetate fraction II and a water fraction. The selective extraction of alkaloids and terpenoids can be achieved in ethyl acetate fraction II [22,37,38] (Figure 1A). Another group performed the extraction of alkaloids from the dried aerial parts of *Haplophyllum sieversii*. The ground plant material was extracted using petroleum ether at room temperature, dried, and subsequently extracted using a hexane/ethyl acetate/H_2_O (54:44:2/*v*:*v*:v) solvent system. This process was again repeated with 95% ethanol and finally with H_2_O [39].

#### 2.2.2. Extraction of Cardiac Glycosides

In one study, each of the plant parts (leaves, stem, fruits, seeds, root, and flowers) was macerated in methanol at room temperature, followed by sonication (20 min). The crude dried extracts were suspended in an aqueous methanolic solution (1:1/*v*:*v*) and fractionated with a separating funnel with n-hexane, followed by chloroform and ethyl acetate [34].

#### 2.2.3. Extraction of Pregnane Glycosides

The dried root bark of *Calotropis gigantea* was utilized for the isolation of bio-active compounds. The root bark of *C. gigantea* was extracted three times with 95% ethanol for 72 h. The ethanolic extract was filtered and concentrated. The ethanolic extract was partitioned between water and n-hexane (1:1/*v*:*v*), separated by a funnel, and the hexane phase was concentrated. The ethyl acetate was added to the water phase and two phases were obtained, i.e., water and ethyl acetate [8] (Figure 1B). The ethyl acetate fraction was used to isolate oxypregnane oligoglycosides. The air-dried aerial parts of *C. quadrangula* were extracted with methanol. The methanol was evaporated and a brown residue was obtained, which was suspended in water and partitioned successively with chloroform and n-butanol to yield three fractions, including a water fraction. The chloroform fraction was used to isolate pregnane oligoglycosides [40]. Zhang et al. utilized the air-dried roots of *Cynanchum otophyllum* for the extraction of bioactive compounds. The dried roots were extracted with ethanol (75%) three times for 3 hours. The organic solvent was removed, and the residue was suspended in water, followed by extraction with CHCl_3_ to afford the CHCl_3_ fraction, which was used to isolate pregnane glycosides [41].

#### 2.2.4. Extraction of Terpenoids

The ground material was extracted with hexane:ethyl acetate (85:15/*v*:*v*); this yielded the crude extract, which was transferred to a glass vial or flask for shaking for at least 3–4 h or overnight for the extraction of terpenoids [36]. Steam distillation was performed on basil leaves and extraction was performed three times with hexane. For ultrasonication, the basil leaves were placed in a vial with the internal standard (dodecane) and hexane. The extraction was performed in an ultrasonic bath at 20 °C for 10 min. For microwave-assisted extraction, the basil leaves were placed in a vessel with hexane and extracted using a microwave extraction system using 40% microwave power for 5 min. After extraction, the vessels were left to cool to room temperature and then kept at 4 °C to avoid the loss of volatile analytes [42]. Some specific chemical methods have been developed to separate terpenoids. When a terpenoid is treated with nitrosyl chloride in chloroform (Tilden’s reagent), crystalline products are obtained and the adducts can be isolated and decomposed into their corresponding hydrocarbons. The diesters can be extracted with NaHCO_3_ and then decomposed by an alkali to yield a terpenoid alcohol. Terpenoid aldehydes and ketones were isolated by reaction with standard reagents such as 2,4 dinitro phenylhydrazine, a phenylhydrazine reagent, etc. Terpenoids can also be isolated by fractional distillation. The hydrocarbon is distilled first, followed by the oxygenated terpenoids [43,44]. The Soxhlet extraction method can be used with petroleum ether to initiate the extraction process. The extraction can be carried out at various temperatures. Hydrodistillation with the Clevenger apparatus can be performed to isolate essential oils from various parts of plants [45]. The leaves of *Camellia japonica* were crushed in liquid nitrogen and mixed with methanol to produce the extract of the leaves, which contained terpenoids [46].

#### 2.2.5. Extraction of Flavonoids

Microwave-assisted extraction (MAE) was performed in a microwave using a closed vessel with pressure. The microwave power, number of extraction cycles, solvent concentration, temperature, and ratio of solvents can be optimized for the extraction of flavonoids [47]. Tobacco leaves were percolated using 70% methanol and the procedure was repeated until a negative cyanidin test confirmed the complete extraction of flavonoids. The methanol was evaporated, and the aqueous extract of flavonoids was partitioned using various solvents. A cyanidin test can be carried out for flavonoids, and ethyl acetate contains the largest amount of flavonoids. Another report evaluated flavonoids and showed that their extraction from pomegranate peel was affected by the pH of the solvent. The best results were observed in an acidic medium, and, at a pH above 7.0, lower extraction yields were recorded [48]. The various extractive methods, such as maceration, percolation, hydrodistillation, and Soxhlet, have evolved [49]. Soxhlet was initially the most common method for the extraction of flavonoids due to its ease of maintenance and lower solvent content compared to other methods [50]. Various solvents have been tested for the extraction of flavonoids, and the yield is affected by a large number of parameters, such as the time, temperature, material–solvent ratio, solvent polarity, etc. [51]. Supercritical fluid extraction (SFE) is another method tested for the extraction of flavonoids. The supercritical fluid generates a gradient of viscosity in the solvent, which improves the transfer of the matter, enhancing the flavonoid yield more significantly compared to other techniques [52,53].

#### 2.2.6. Extraction of Phenolic Compounds

The ripe fruits of the *Myristica* species were extracted with ethanol in an ultrasonic water bath (53 KHz for 30 min) at room temperature. After 24 h, the extract was filtered, and the residue of each sample was further extracted thrice with the same procedure. The dried residue of each sample was weighed and dissolved in methanol/chloroform (50:50/*v*:*v*) for 1 h, filtered, and yielded the phenolic compounds [7].

### 2.3. Characterization of Secondary Metabolites

**Figure 2:**
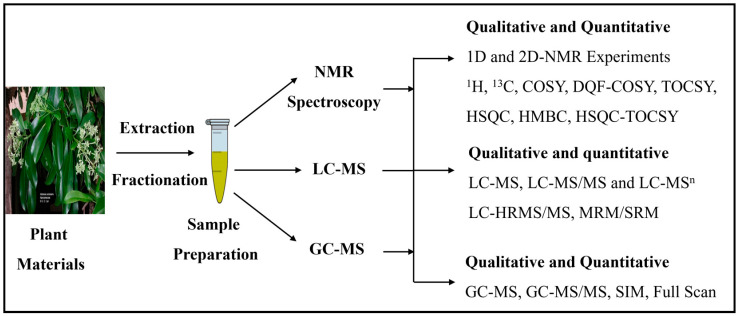
Schematic diagram for analysis of plant-derived mixtures: qualitative (chemical structural information) and quantitative analysis can be performed using NMR, LC-MS, and GC-MS techniques.

The secondary metabolites extracted using various methods need to be characterized properly to identify known compounds and discover new compounds. The exact chemical composition of the mixture can be determined with the help of various analytical techniques. The combination of various analytical techniques or individuals can be used to profile all of the chemical constituents present in the mixture (Figure 2).

#### 2.3.1. Liquid Chromatography–Mass Spectrometry (LC-MS)

The integration of liquid chromatography (LC) with high-resolution mass spectrometry (HRMS), and tandem mass spectrometry (MS/MS) is a powerful tool to analyze or identify a wide range of compounds in various complex mixtures [7,54]. The chromatographic method needs to be optimized for the satisfactory separation of compounds, resulting in the best-quality LC-MS data. For phenolic compounds, Renu et al. utilized the C_18_ column (10 cm × 2.1 mm, 2.7 µm). Moreover, different mobile phase systems and flow rates were compared to achieve better separation and ionization. The systems included water–acetonitrile (ACN), 0.1% formic acid in water–ACN, water–MeOH, and 0.1% formic acid in water–MeOH at different flow rates. A mobile phase of 0.1% formic acid in water and MeOH at a flow rate of 0.3 mL/min was found to be the best for phenolic compounds. The compounds, dependent on the multiple reaction monitoring (MRM) parameters and ionization source parameters, were optimized to achieve the optimal characteristics and a stable multiple reaction monitoring transition for each analyte [7]. Rohit et al. recorded ESIMS and LC-MS data for oxypregnane oligoglycosides on the LCQ ion trap and AQUITY TQD mass spectrometers. HRMS was recorded on a Q-TOF mass spectrometer for the same class of compounds [8]. For the chromatographic separation of cardiac glycosides (CGs), 5 mM NH_4_CH_3_CO_2_ buffer was prepared in an aq. acetonitrile solution (95:5/*v*:*v*) with a pH of 5.53. The separation and analysis of the CGs was carried out on a UPLC–ESI/TQD-MS system with the help of an ethylene-bridged hybrid BEH C_18_ (100 mm × 2.1 mm, 1.7 μm) reverse-phase column. The UPLC eluent was subjected to the mass spectrometer, equipped with an electrospray ionization (ESI) source, operated in dual ion mode. Argon gas was used as a collision-induced dissociation (CID) gas, and the collision cell energy was applied in a linear ramp from 30 to 5 eV [34].

#### 2.3.2. Nuclear Magnetic Resonance (NMR) Spectroscopy

NMR spectroscopy is a widely used technique for the structure elucidation of compounds in isolated as well as complex mixtures (plants as well as animals) [7,8,22,27]. The characterization of various secondary metabolites can be performed using 1D and 2D NMR experiments. Multiplicity information cannot be efficiently collected in complex mixtures or natural product extracts, due to overlapping signals. Alternatively, 2D-JRES can be used to achieve homonuclear decoupled spectra [55]. Computer-assisted structure elucidation (CASE) has been developed for chemical structure elucidation using 1D and 2D NMR data [56]. Various 2D NMR experiments, such as correlation spectroscopy (COSY), total correlation spectroscopy (TOCSY), double quantum filtered correlation spectroscopy (DQF-COSY), ^1^H-^13^C heteronuclear single quantum correlation (HSQC), and, for the connectivity of groups or rings, ^1^H-^13^C heteronuclear multiple bond correlation (HMBC) experiments, can be used. An advanced variant of HSQC, namely multiplicity-edited HSQC, could be used for structure characterization in a mixture [22]. Five NMR pulse sequences can be combined into one supersequence, and this can dramatically reduce the analysis time for basic NMR applications [57]. The use of NMR by ordered acquisition using ^1^H detection (NOAH) can combine multiple NMR experiments in theory, leading to thousands of plausible supersequences [58] Diffusion-ordered spectroscopy (DOSY) NMR is a useful tool for the analysis of complex mixtures [59]. DOSY NMR and chemometrics were developed to determine the molecular weights of natural polymers [60]. Additionally, LC-MS-NMR strategies have been developed for the separation and characterization of metabolites in complex biological mixtures [54].

#### 2.3.3. Gas Chromatography–Mass Spectrometry (GC-MS)

GC-MS is one of the most widely utilized chromatographic techniques for the high-throughput screening of complex mixtures [61,62,63]. GC-MS can detect and quantify lipophilic metabolites including fatty acids, alkanes, sterols, phytohormones, and tocopherols [62,63]. Thermally stable and volatile compounds/derivatized compounds can be analyzed by GC-MS. The hyphenated technology using a gas chromatograph and mass spectrometer can be uniquely suited for mixture analysis. Helium is the most preferable carrier gas and the ionization source is an electron impact ionization device with 70 eV, making it a universally acceptable tool with consistent results for a wide range of biological samples [30,42].

## 3. Results

**Figure 3:**
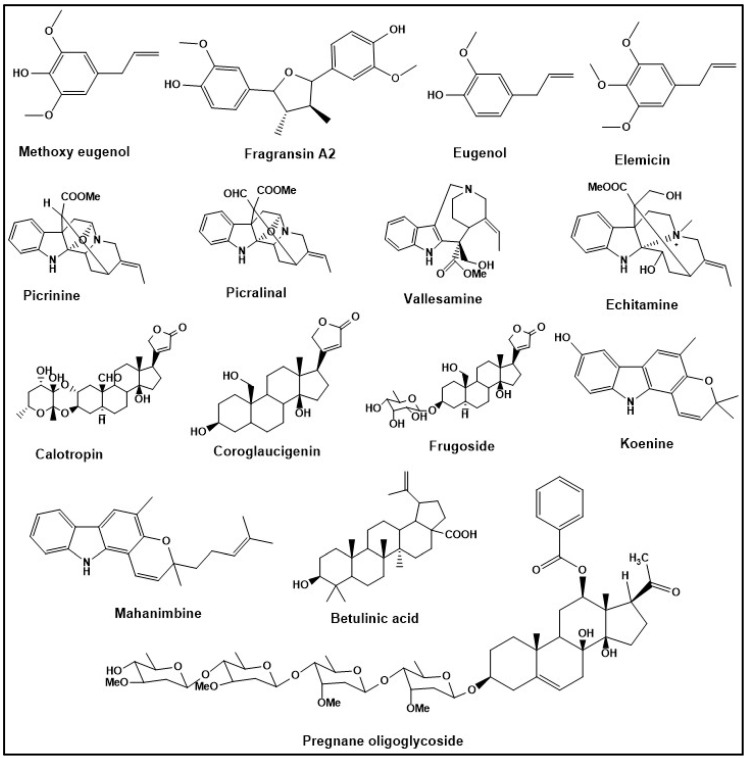
Structures of classes of compounds: representative compounds from some of the plant-derived bioactive compounds, showing the chemical structural diversity in nature.

During the extraction of alkaloids, the first partitioning of the acidic solution with ethyl acetate results in the green-colored fraction of ethyl acetate, which might contain chlorophyll and other pigments, and non-polar compounds. In one study, basifying the acidic solution, followed by partitioning with ethyl acetate, resulted in a fraction of alkaloids and terpenoids containing carboxylic acid groups [22] (Figure 1A). The structures of some of the representative compounds of alkaloids and terpenoids, such as picrinine, picralinal, koenine, mahanimbine, vallesamine, echitamine, and betulinic acid, are shown in Figure 3. Another method utilizing ethyl acetate for partitioning resulted in a fraction containing aglycone–glycone conjugate molecules [8] (Figure 1B). Some of the representative compounds of these types of classes are calotropin, frugoside, pregnane oligoglycosides, and coroglaucigenin (Figure 3). LC-MS with an ESI source provides the product ion information with various adducts, such as [M+H]^+^, (M-H]^+^ [M+NH_4_]^+^, and [M+Na]^+^, etc., in both ionization modes, which is useful for molecular mass determination. The MS/MS fragmentation pattern indicates the presence of aglycone (steroid or genin) moieties in natural products, such as cardiac glycosides, pregnane glycosides, and oxypregnane oligoglycosides. This is a powerful technique to reveal the loss of sugar moieties and also indicates glycosidic linkages between two sugar molecules [34].

**Figure 4:**
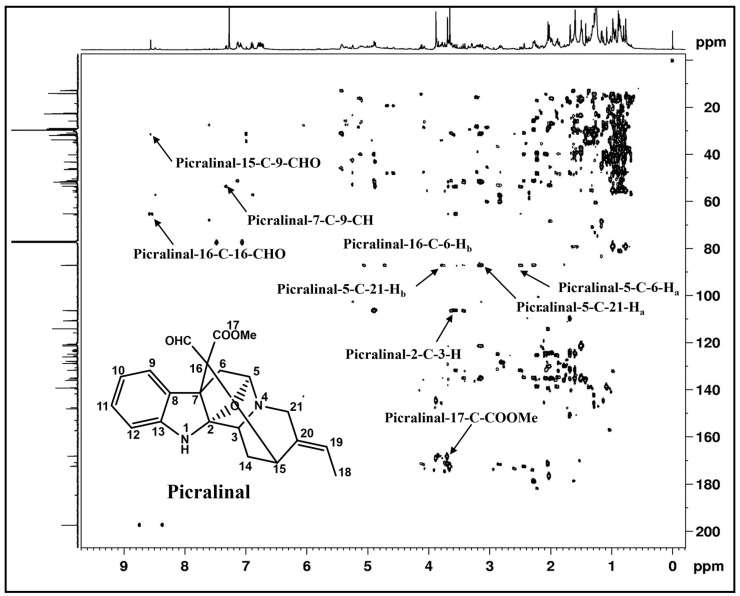
Representative HMBC spectrum of alkaloid picralinal: ^1^H-^13^C multiple bond correlations showing the connectivity of the carbon skeleton in picralinal. Its detailed characterization is described in a previous paper [22].

Additionally, many natural products show the characteristics of chemical shift patterns in NMR spectroscopy. Glycosides have characteristic ^13^C NMR signals for anomeric carbon signals. TOCSY 2D NMR is a remarkable technique to demonstrate the spin system of sugar molecules and edited 2D HSQC to reveal the CH_2_ (methylene) signals in negative ^1^H-^13^C correlations [8]. Ammoniated adduct [M+NH_4_]^+^ was prominently found as compared with [M+H]^+^ under positive ESI mode, while the [M-H]^−^, [M+Cl]^−^, and [M+CH_3_COO]^−^ adducts were also observed in negative ESI mode. The in-source fragmentation pattern and tandem mass analysis generated product-ion-containing structural information about cardiac glycosides. It showed significant m/z peaks depicting the cleavage of an O-glycosidic bond, revealing the presence of mono-, di-, or triglycones linked with aglycone moieties [34]. Renu et al. showed that the MS/MS fragmentation pattern identified compounds tentatively, which was strongly supported by 1D and 2D NMR spectroscopy for absolute structure elucidation, even in a complex mixture [7]. Rohit et al. utilized homonuclear and heteronuclear 2D NMR experiments to identify alkaloids in the alkaloidal fraction of the different parts of *Alstonia scholaris* [22]. Moreover, 2D correlation spectroscopy (COSY) is a crucial method; it is a proton-detected technique and provides information about J-coupled protons. The ^1^H NMR spectrum is shown on both axes, with a cross-peak for J-coupled protons. The HSQC spectrum elucidates the directly attached (or coupled) protons for specific carbons or heteroatoms in the chemical structure through covalent bonds. The HMBC experiment provides information on correlated carbons and protons that are connected by two, three, and four bonds in the backbone of the same molecule (Figure 4) [7,22,33]. This is an excellent tool for the determination of the positions of quaternary carbon atoms in complex molecules.

## 4. Discussion

Phytochemicals are plant origin natural products and play an important role in ensuring the health status of plants. These natural products have been utilized continuously in animal experiments, cell and tissue cultures, and human clinical trials for therapeutic application against various diseases. Natural products can be classified into primary metabolites, which are essential for an organism's growth, and secondary metabolites, which are essential for survival within the environment and against herbivores [64,65]. Natural products can be biosynthesized by primary metabolites, i.e., alkaloids derived from primary metabolites that contain nitrogen, such as amino acids, the polyketides synthesized from acetate and malonate, and the terpenoids synthesized from isoprene (five-carbon building block).

As only > 10% of the global biodiversity has been examined for its therapeutic potential, many more useful plant-derived lead molecules await discovery, but there are many challenges involved in accessing this naturally occurring chemical diversity [66]. Scientists have devoted much effort to selectively extracting compounds from natural sources. For example, alkaloids can be specifically isolated by acidifying the solution, as hydrochloric acid (HCl) converts the basic functional groups of alkaloids into chloride salts, followed by basifying the solution, which could be helpful to selectively extract alkaloids. The mid-polar compounds can be extracted with efficiency in mid-polar solvents such as ethyl acetate, as well as chloroform through successive partitioning with highly non-polar (e.g., hexane) and polar (e.g., water) solvents. The conventional methods are safe for thermosensitive compounds, but the usage of large volumes of solvents and long extraction times are disadvantageous, motivating the development of newer methods for extraction. The solvents play an important role in isolating the class of compounds, e.g., the extracted lipids by non-polar solvents were found to be more numerous than those extracted with polar solvents. In contrast, amino acids and sugars can be isolated in larger amounts by polar solvents or a mixture of polar and mid-polar solvents [67]. The polarity-dependent increase in antioxidant activity indicates the extraction of more polar and strong antioxidant compounds such as phenolic and flavonoids in polar solvents [68]. The MAE and UAE methodologies are quite useful as the extraction efficiency is high, with minimal organic solvent use, and less time for extraction is required, but the radiation-sensitive compounds can be damaged. Each of the extraction methods has its advantages and disadvantages, which are listed in Table 1.

Technological advancements have increased the efficiency of analytical techniques to identify known and unknown compounds in complex mixtures. Ultraperformance liquid chromatography (UPLC) in conjunction with ESI tandem mass spectrometry is one of the most advanced, fast, and economical tools in natural product research, used to investigate secondary metabolites, chemical diversity exploration, and tentative structure elucidation [69]. The absolute chemical structure can be determined with the help of extensive 1D and 2D NMR spectroscopy [70,71]. Homonuclear NMR experiments such as COSY and TOCSY can establish the spin systems of the molecules in a mixture [48]. The disadvantage of COSY is that the signals can be overlapped for similar types of structural backbones, and the further 2D NMR experiments need to be optimized. Heteronuclear correlation (^1^H-^13^C) experiments can play a huge role in the structure elucidation of several compounds in a given mixture, as the resolution in the second dimension (^13^C) of 2D NMR is an advantage. The correlation in the 2D NMR spectrum can be well dispersed for several components and can be analyzed precisely. Distortionless enhancement by polarization transfer (DEPT) could provide multiplicity information about carbon as DEPT-135 provides information via CH and CH_3_ up and CH_2_ down in the spectrum, but it does not provide proton chemical shift information as it is only focused on carbon chemical shifts. HSQC provides chemical shifts for both protons and carbons with their direct connectivity. Multiplicity-edited HSQC shows CH and CH_3_ signals in one color contour (or up), with CH_2_ in another color contour (or down). Edited HSQC displays the number of protons attached to each carbon and correlations between the chemical shifts of both types of atoms. Another advantage of this experiment is the acquisition of information on chemical shift correlations and multiplicity in significantly less time than in a carbon-based experiment.

Another excellent tool for mixture analysis is ^1^H-^13^C HSQC-TOCSY, which combines HSQC with TOCSY to give through-bond correlations of ^1^H-^13^C from all other coupled protons to that proton. The coupled protons can be observed in the same ^13^C chemical shift of the carbon atom coupled to the primary proton [7,22]. A DOSY experiment exclusively separates the NMR signals of different chemical species according to their diffusion coefficients, which is useful in determining the molecular weight of compounds. This technique is quite beneficial in the study of secondary metabolites in complex mixtures [59].

GC-MS could provide the highest resolution for the separation of compounds with less method development, as a single gaseous mobile phase is used with a temperature gradient. Furthermore, GC-MS with electron impact ionization provides a fingerprint of the primary and secondary metabolites, making GC-MS an indispensable tool in mixture analysis [72,73]. Selective extraction followed by the characterization of secondary metabolites with the help of sophisticated instruments is highly important in identifying the active principles in plant-derived extracts. The advantages and disadvantages of various techniques are summarized in Table 2.

## 5. Conclusions and Future Directions

To obtain the best biological activity from a class of compounds, a selective extraction procedure is highly needed. Partitioning through an ethyl acetate solvent provides the best extraction efficiency for aglycone and glycone conjugate moieties (cardiac glycosides, pregnane glycosides, etc.). The acid–base-guided solvent procedure could be the best extraction procedure for the isolation of alkaloids in a specific fraction. The methods summarized under each class of compounds can be generalized for various types of plants provided that the researcher is interested in a specific class of compounds. The wide polarity range of molecules covered by LC-MS/MS makes this technique unique for the qualitative assessment of secondary metabolites in a mixture [74]. GC-MS could provide a higher resolution, but derivatization is needed for polar compounds in order to analyze them under GC-MS. However, steroids, polyaromatic hydrocarbons, and volatile and thermally stable compounds can be analyzed directly. The advantages of the chemical shifts and inherent resolution in ^13^C-NMR enhance the capability of homo- and heteronuclear 2D NMR spectroscopy for structure elucidation in complex mixtures [75]. This manuscript provides a basic understanding of the extraction and characterization of diverse classes of compounds in mixtures.

## Figures and Tables

**Table 1:** Comparison of extraction methods for isolation of different classes of compounds. Note: the crude extracts obtained from these methods can be further used for the isolation of various classes of compounds through various solvent–solvent partitioning and acid–base solution methods.

Methods	Advantages	Disadvantages
Maceration	It is a simple extraction method with minimal set-up needed. It can be used for the extraction of thermolabile metabolites. It can be considered economically beneficial due to the lack of heat.	Long extraction time and low extraction efficiency. Large volume of extraction solvent is required.
Percolation	Percolation is efficient as it is a continuous process in which the saturated solvent is replaced with fresh solvent constantly.	Long extraction time and low extraction efficiency. Large volume of extraction solvent is required.
Soxhlet extraction	Simple method to set up. Temperature in the extraction system can be maintained. Temperature could help to rupture the plant tissues and the metabolites can be extracted with greater efficiency.	Requires excessive extraction time. Uses large amounts of solvents. No agitation is applied to accelerate the process. Heat-sensitive compounds can be thermally decomposed.
Sequential extraction method	Methodological simplicity as it involves subsequential addition and removal of solvents. Simple apparatus required.	Labor-intensive and consumption of large volume of solvents. It can be environmentally hazardous as many solvents are used. Low selectivity and handling of large sample volumes.
Microwave-assisted extraction	Moderate or no volume of organic solvent consumed. Applicable for both industrial and laboratory scales. Less time-consuming than conventional methods. High efficiency as it changes the cell structure due to electromagnetic waves.	Efficiency of MWE is very poor for non-polar compounds or solvents. Less efficiency for extremely viscous solvents. Not appropriate for heat-sensitive organic compounds. Expensive instrumental set-up and difficult to operate.
Ultrasound-based extraction	Moderate volume of organic solvent consumed.Less extraction time. Less damage to bioactive compounds. Uniform distribution of energy enhances extraction efficiency.	Expensive set-up and requires optimization. It can cause some unwanted changes to bioactive compounds.

**Table 2:** Comparison of the techniques for characterization of the compounds in complex mixtures. Note: these techniques can be used individually and in combination to characterize the compounds in mixtures.

Technique	Advantages	Disadvantages
NMR	Chemical shift advantage provides information about various groups in compounds. Qualitative and quantitative analysis of compounds can be performed in mixtures. Extensive 1D and 2D NMR experiments help to elucidate the structures of compounds. Minimal sample preparation and no method development required once optimized.	Low sensitivity for some of the NMR active nuclei (i.e., ^13^C) due to low gyromagnetic ratio and very low natural abundance. Low resolution in proton NMR spectroscopy prohibits identification of overlapped signals. High magnetic field instruments are costly, but resolution could be enhanced.
LC-MS	Positively and negatively charged adduct ions formed with atoms or molecules can help to determine the exact molecular masses of compounds. Linear dynamic range with low detection limit. Capability to quantify multiple analytes simultaneously. MS/MS fragmentation pattern is very helpful in characterizing the compounds.	Extensive method development needed based on the class of compounds to be analyzed. Testing of several columns for different classes of compounds. High purchase, maintenance, and operational costs. Different ionization sources in mass spectrometer are required for different polarities of compounds.
GC-MS	High resolution power and higher sensitivity compared to other methods. GC-MS has high accuracy and precision and can resolve closely related compounds. Small sample volume can be separated using gas chromatography. Fragmentation pattern provides unique fingerprint for each chemical structure.	GC-MS is limited to volatile compounds. Compounds can decompose at high temperatures. Thermal stability is necessary for separation through gas chromatography. It is not suitable for high-boiling-point and polar analytes. Chemical derivatization is needed to make compounds volatile.

## Data Availability

Not applicable.
